# An *in vivo *Method for Determination of Endosomal Distribution of Both Ligand and Asialoglycoprotein Receptor in Rat Liver

**DOI:** 10.1186/1476-5926-2-S1-S38

**Published:** 2004-01-14

**Authors:** Shana R Dalton, Robert L Wiegert, Carol A Casey

**Affiliations:** 1 University of Nebraska Medical Center and Department of Veterans Affairs Medical Center, Omaha, NE, USA

## Introduction

Alcoholic liver injury is a significant medical problem throughout the world. Protein trafficking pathways, including endocytosis, appear to be especially susceptible to the deleterious effects of alcohol. Using the asialoglycoprotein receptor (ASGPR) as a model, we have studied ethanol-induced alterations in the process of receptor-mediated endocytosis (RME). The ASGPR, a hepatocyte-specific receptor, binds proteins that have lost their terminal sialic acid moiety and have exposed galactose or N-acetylgalactosamine moieties [1]. During RME many extracellular ligands are bound by specific cell surface receptors, such as the ASGPR, and internalized via a clathrin-coated pit pathway [2]. In the endocytotic pathway, the lumen of the endosome becomes acidic allowing the receptor-ligand complexes to dissociate. The separated receptors and ligands can either be targeted for degradation in lysosomes or recycled back to the cell surface. Endosomal trafficking from the cell surface to the final destination in the cell is an extremely complex process that is still not completely understood.

Our previous studies using the ASGPR model have identified several ethanol-induced alterations during the process of RME. Altered receptor-ligand uncoupling and endosomal acidification as well as decreased ligand binding, internalization, and degradation are some of the observed alterations [3-9]. For the current study, we wanted to examine the trafficking/distribution of both ligand and receptor in liver endosomes. To accomplish this we labeled asialoorosomucoid (ASOR), a ligand for the ASGPR, with either Texas-Red (TR-ASOR) or radioactive iodine (^125^I-ASOR). We exposed rats *in vivo *to the labeled ligand, and then assessed both ligand and ASGPR content in two isolated liver endosome populations. The "early endosomes" (EE) consist of endosomes from the periphery of the cell, including endosomes just entering the pathway and those containing receptor and/or ligand being recycled back to the cell surface. The "late endosomes" (LE) contain more distal endosomes which are destined for degradation in lysosomes. The purpose of the work described here was to gather information on the viability of this method for isolating endosome fractions and for assessing both ligand and receptor content in the isolated endosome fractions. Ultimately, our goal is to use this method to further investigate the affect of ethanol on the trafficking of ligand and receptor between endosome fractions.

## Methods

Desialyation and iodination of orosomucoid was conducted using techniques previously described and standard to our laboratory [3]. Labeling of ASOR with Texas Red was done using the FluoReporter^– ^Texas Red^–^-X protein labeling kit (Molecular Probes, Eugene, OR). Male Wistar chow-fed rats (200–250 g) were used for the current studies. Labeled ligand was injected via the penile vein; and either 5 min or 25 min after injection, the liver was perfused with ice cold KRH/10 mM EDTA, pH 7.4 to remove any surface bound ligand, flush blood from the liver, and stop endocytosis. Livers were removed and homogenized in 5 volumes (wt/v) of ice cold homogenization buffer (3 mM imidazole, 0.25 M sucrose), pH 7.4 in the presence of Sigma protease inhibitor cocktail. Liver homogenates were processed to obtain a microsomal/endosomal pellet that was resuspended in 1.5 M sucrose and applied to the bottom of a discontinuous sucrose gradient. After centrifugation (62,500 – g, 3 hr, 4 degrees C), two endosome fractions were isolated from the sucrose gradient. The fraction enriched in EE was obtained from the 0.86 M/1.15 M interface, and the LE enriched fraction was obtained from the 0.6 M/0.86 M interface. The amount of TR-ASOR or ^125^I-ASOR was quantified for each time point. For Western blot analysis, protein concentration was adjusted to 2–5 mg/ml in Laemmli sample buffer and fractionated on 10% SDS-PAGE gels. Protein was then transferred to nitrocellulose membrane, and the blots exposed to one of the primary antibodies. Primary antibodies included a monoclonal mouse anti-actin (Chemicon, Temecula, CA), polyclonal rabbit anti-Rab 4 (Santa Cruz Biotech, Santa Cruz, CA), and a polyclonal rabbit anti-ASGPR produced in our laboratory [7]. After incubation with the appropriate secondary antibody, the immunoreactive proteins were visualized colorimetrically and quantified by scanning densitometry.

## Results and Discussion

For the current study, we wanted to isolate two endosome populations: one enriched in "early endosomes" and the other in "late endosomes." Pol and coworkers [10-12] have isolated three distinct endosome populations: the compartment of uncoupling receptor and ligand (CURL), considered the early endosomes, the multivesicular bodies (MVBs), considered the late endosomes, and the receptor-recycling compartment (RRC), which may recycle from the early or the late endosomes. For this study, the endosomes that were isolated compare to the above endosome fractions as follows: the fraction we termed EE consists of the two most dense endosome fractions, the RRC and CURL, and our LE fraction consists of the less dense MVBs.

Separation of the endosome fractions was assessed using Western blot analysis for the early endosome markers, actin and Rab 4. The endosome samples exposed to the anti-actin antibody showed a strong presence of actin in the EE fractions compared to the LE fractions, where only negligible amounts were detected. The same pattern was observed when the endosome fractions were exposed to the anti-Rab 4 antibody. These results indicate that isolation of the endosomes into fractions enriched in either "early" or "late" endosomes was accomplished. As expected, more ligand was internalized at the 25 min post-injection time point compared to the 5 min post-injection time point. In order to compare the distribution patterns at the two post-injection time points, both ligand and ASGPR content of the different endosome fractions was assessed as a percentage of the total content in the whole endosome population. At both time points, we observed that the EE fraction had significantly more ligand compared to the LE fraction (p – 0.001), and the same endosomal distribution pattern was observed for the ASGPR. These results indicate that following an *in vivo *injection of labeled ligand it is possible to determine the distribution pattern of ligand in two distinct endosome fractions. In addition, the distribution pattern of receptor can also be assessed in isolated endosomes.

Collectively, the results presented here indicate that the isolation of two distinct endosome populations, as well as the characterization of both the ligand and receptor content of the isolated endosomes is possible. Using whole cell hepatocyte preparations we have previously identified ethanol-induced impairments in ligand binding, internalization, and degradation, receptor recycling, and endosome acidification [3-6]. Two different time points were chosen for the studies discussed here. The first time point was at 5 min post-injection which was early in the process of endosome trafficking. The second time point, 25 min post-injection, was chosen because it was approximately midway in the process. The latter time point will be particularly important in studies that involve ethanol-exposed animals since it should allow for the visualization of any ethanol-induced alterations in the endosomal distribution of ligand and receptor. As stated earlier, our ultimate goal was to use this *in vivo *method to investigate the affect of ethanol on the trafficking of ligand and receptor between endosome fractions. Studies are currently underway using ethanol-fed animals and the method described above, and preliminary results indicate that exposure to ethanol alters the endosomal distribution of both ligand and ASGPR.

## References

[B1] Ashwell G, Harford J (1982). Carbohydrate-specific receptors of the liver. Annu Rev Biochem.

[B2] Goldstein JL, Brown MS, Anderson RG, Russell DW, Schneider WJ (1985). Receptor-mediated endocytosis: concepts emerging from the LDL receptor system. Annu Rev Cell Biol.

[B3] Casey CA, Kragskow SL, Sorrell MF, Tuma DJ (1987). Chronic ethanol administration impairs the binding and endocytosis of asialo-orosomucoid in isolated rat hepatocytes. J Biol Chem.

[B4] Casey CA, Kragskow SL, Sorrell MF, Tuma DJ (1990). Effect of chronic ethanol administration on total asialoglycoprotein receptor content and intracellular processing of asialoorosomucoid in isolated rat hepatocytes. Biochim Biophys Acta.

[B5] Casey CA, Camacho KB, Tuma DJ (1992). The effects of chronic ethanol administration on the rates of internalization of various ligands during hepatic endocytosis. Biochim Biophys Acta.

[B6] Casey CA, Wiegert RL, Tuma DJ (1993). Chronic ethanol administration impairs ATP-dependent acidification of endosomes in rat liver. Biochem Biophys Res Commun.

[B7] Tworek BL, Tuma DJ, Casey CA (1996). Decreased binding of asialoglycoproteins to hepatocytes from ethanol-fed rats. J Biol Chem.

[B8] Tworek BL, Wiegert RL, Jeanette JP, Tuma DJ, Casey CA (1998). Differential effects of monensin on asialoglycoprotein receptor function after short-term ethanol administration. Biochem Pharmacol.

[B9] Tworek BL, Wiegert RL, Tuma DJ, Casey CA (1998). Effects of monensin on ethanol-induced alterations in function of hepatocellular asialoglycoprotein receptor subpopulations. Alcohol Clin Exp Res.

[B10] Pol A, Enrich C (1997). Membrane transport in rat liver endocytic pathways: preparation, biochemical properties and functional roles of hepatic endosomes. Electrophoresis.

[B11] Pol A, Ortega D, Enrich C (1997). Identification of cytoskeleton-associated proteins in isolated rat liver endosomes. Biochem J.

[B12] Pol A, Ortega D, Enrich C (1997). Identification and distribution of proteins in isolated endosomal fractions of rat liver: involvement in endocytosis, recycling and transcytosis. Biochem J.

